# Route of Administration of the TLR9 Agonist CpG Critically Determines the Efficacy of Cancer Immunotherapy in Mice

**DOI:** 10.1371/journal.pone.0008368

**Published:** 2009-12-18

**Authors:** Stefan Nierkens, Martijn H. den Brok, Thijs Roelofsen, Jori A. L. Wagenaars, Carl G. Figdor, Theo J. Ruers, Gosse J. Adema

**Affiliations:** 1 Department of Tumor Immunology, Radboud University Nijmegen Medical Centre, Nijmegen, The Netherlands; 2 The Netherlands Cancer Institute, Amsterdam, The Netherlands; University Hospital Zurich, Switzerland

## Abstract

**Background:**

The TLR9 agonist CpG is increasingly applied in preclinical and clinical studies as a therapeutic modality to enhance tumor immunity. The clinical application of CpG appears, however, less successful than would be predicted from animal studies. One reason might be the different administration routes applied in most mouse studies and clinical trials. We studied whether the efficacy of CpG as an adjuvant in cancer immunotherapy is dependent on the route of CpG administration, in particular when the tumor is destructed *in situ*.

**Methodology/Principal Findings:**

In situ tumor destruction techniques are minimally invasive therapeutic alternatives for the treatment of (nonresectable) solid tumors. In contrast to surgical resection, tumor destruction leads to the induction of weak but tumor-specific immunity that can be enhanced by coapplication of CpG. As in situ tumor destruction by cryosurgery creates an instant local release of antigens, we applied this model to study the efficacy of CpG to enhance antitumor immunity when administrated via different routes: peritumoral, intravenous, and subcutaneous but distant from the tumor. We show that peritumoral administration is superior in the activation of dendritic cells, induction of tumor-specific CTL, and long-lasting tumor protection. Although the intravenous and subcutaneous (at distant site) exposures are commonly used in clinical trials, they only provided partial protection or even failed to enhance antitumor responses as induced by cryosurgery alone.

**Conclusions/Significance:**

CpG administration greatly enhances the efficacy of in situ tumor destruction techniques, provided that CpG is administered in close proximity of the released antigens. Hence, this study helps to provide directions to fully benefit from CpG as immune stimulant in a clinical setting.

## Introduction

Surgical resection of solid tumors generally offers the best chance of cure in cancer patients. In many occasions however tumor lesions are not eligible for resection and require alternative destruction techniques, such as cryosurgery, radiofrequency or laser ablation. These methods offer minimally invasive treatments for a large range of tumors. In fact, radiofrequency was found to provide local tumor control equivalent to resection in a subgroup of patients [1], emphasizing that in situ tumor destruction techniques become increasingly successful treatment modalities.

In contrast to surgical resection, in situ tumor destruction provides an antigen source available for immune cells. The involvement of the immune system in the clearance of tumor cells is increasingly appreciated [2]. Antigen-presenting cells (APC), such as dendritic cells (DC), are well-equipped to phagocytose dying cells and process tumor antigens for presentation to T lymphocytes [3]. Indeed, recent data demonstrated that DC in the tumor draining lymph nodes efficiently acquire tumor debris following in situ tumor destruction [4]. Unfortunately, although tumor ablation has been associated with the occurrence of immune activation in some patients [5], the outgrowth of distant micrometastases implies that no or weak systemic protective immune responses are induced.

Applying a recently developed mouse model we previously showed that in situ tumor destruction by means of cryosurgery or radiofrequency ablation led to the induction of weak but tumor-specific immunity [6], [7]. These results implied that the combination of in situ tumor destructive treatment modalities with specific immune stimulation could be a strategy to enhance the clinical outcome for an increasing number of cancer patients.

In this respect, toll-like receptor (TLR) agonists are of great interest as immune adjuvants. TLR are a family of pathogen recognition receptors that are triggered upon recognition of pathogen-associated molecular patterns expressed by a diverse group of infectious microorganisms. Interestingly, the immunostimulatory potency of Bacille Calmette-Guerin (BCG), already used in the 1970s to stimulate antitumor immunity [8], is now known to be BCG DNA that binds to TLR9 [9], [10]. Further studies demonstrated that DC express TLR9 and are directly activated through binding of TLR9 to unmethylated CpG motifs in DNA [8]. DC activation by CpG involves a signaling cascade culminating in the activation of transcription factors e.g. nuclear factor-κB (NF-κB), subsequent upregulation of co-stimulatory molecules and production of chemokines and cytokines (e.g. IL-6, IL-12, TNF-α). As a result, CpG-stimulated DC induce Th1 responses and are instrumental for the activation of CD8^+^ cytotoxic T lymphocytes (CTL).

Indeed, we previously reported that antitumor responses were synergistically enhanced when CpG was used as an adjuvant in the cryo ablation model. Co-injection of CpG increased the formation of tumor-specific CD8^+^ CTL and protected ∼100% of the mice against a re-challenge with tumor cells in this particular model [6]. In other models, CpG has been shown efficient in preventing tumor outgrowth in a prophylactic setting and also eradicated established tumors in mice [11], [12]. The application of CpG in clinical trials appears however less successful than would be predicted from animal studies. A common argument used is the differential expression of TLR9 in DC between mice and man. TLR9 is abundantly expressed in plasmacytoid DC (pDC) from mouse and man, and in murine myeloid DC (mDC). For human mDC, TLR9 expression is less clear as some studies reported weak to negative expression [13], [14], while a recent study shows that human mDC do contain TLR9 protein in amounts comparable with pDC [15]. Moreover, mDC and pDC appear to be functionally linked in both species as intensive cross-talk between the DC subsets is essential for CpG-induced immune activation in both mice and man [16], [17].

We noted that in murine studies, showing the most potent CpG effects, CpG is provided along with the antigen or in close proximity to tumor antigens [12], [18], [19], [20]. Moreover, we have recently reported that co-localization of antigen and CpG within the DC's endosomal compartments in vivo is closely associated with the capability to cross-present antigen and the induction of subsequent protective immunity after ablation [21]. In clinical studies the route of CpG administration is seldom based on the location of the tumor but is generally directed by the supplier's regulations and/or the ease of administration. As these differences in CpG administration routes may have major implications for its clinical efficacy, we compared the efficacy of CpG administered via different routes relative to the site of antigen availability in a unique murine tumor ablation model. Our data show that the intravenous (i.v.) and subcutaneous (s.c.) routes of CpG administration (mostly applied in the human setting) are far less effective than peritumoral (p.t.) CpG injections. We conclude that the failure or success of CpG administration critically depends on the location of CpG administration relative to the location of the tumor.

## Materials and Methods

### Mice and Tumor Cells

C57BL/6n mice (6–8 weeks old) were purchased from Charles River Wiga (Sulzfeld, Germany) and maintained under specific pathogen-free barrier conditions at the Central Animal Laboratory (Nijmegen, The Netherlands). Drinking water and standard laboratory food pellets were provided ad libitum and mice were allowed to settle for at least 1 week before random assignment into specific treatment groups. The experiments were performed according to the guidelines for animal care of the Nijmegen Animal Experiments Committee.

The OVA-transfected murine melanoma cell line B16F10 (B16OVA, clone MO5) was kindly provided by dr. K. Rock [22] and cultured in complete medium (MEM, 5% fetal bovine serum (Greiner Bio-one), 100 U/ml penicillin G sodium and 100 µg/ml streptomycin (Pen/Strep), MEM sodium pyruvate (1 mM), NaH_2_CO_3_, MEM vitamins, MEM non-essential amino acids (all from Invitrogen), 20 µM β-mercaptoethanol (β-ME)) supplemented with 30 µg/ml hygromycin and 1 mg/ml G418. The B16F10 melanoma cell line was obtained from the American Type Culture Collection and maintained in complete medium.

### Tumor Model and Cryosurgery

Tumor cells were suspended in a mixture of PBS and Matrigel (2∶1) and 0.5*10^6^ cells in a total volume of 50 µl were injected s.c. at the right femur. When tumor diameters measured between 6–8 mm (generally at day 9–10), mice were randomly assigned to treatment groups (non-treated; NT, cryo ablation; cryo). Cryo ablation was performed under isoflurane/O_2_/N_2_O anaesthesia using a liquid nitrogen cryo ablation system (CS76, Frigitronics, Shelton, CT) of which the tip is cooled by a continuous flow of circulating liquid nitrogen. During 2 treatment cycles of freezing and thawing the tumor was macroscopically frozen, while leaving surrounding healthy tissue intact. To monitor the induction of long-lasting tumor protection, mice were re-challenged with 25*10^3^ B16OVA cells 40 days after cryo ablation. Two months later, mice that survived the first tumor re-challenge received a second re-challenge with 5*10^4^ B16OVA or B16F10 cells. Mice were sacrificed when tumor volume exceeded 1000 mm^3^ or when tumors brake through the skin barrier.

CpG 1668 (′5-TCCATGACGTTCCTGATGCT-3′) with phosphorothioated backbone was purchased from Sigma Genosys (Haverhill, UK). CpG was injected in PBS p.t. (100 µg divided over 3 injections of 10 µl surrounding the tumor), i.v. (100 µg in 100 µl), or s.c. (100 µg in 30 µl on the left femur of the mouse, i.e. on the contra lateral side of the mouse referred to the location of the tumor). Injections were performed within 30 minutes after ablation. We used CpG 1668, which is a type B CpG, similar to the clinical-grade available CpG currently used in clinical trials.

### Enrichment and Analyses of DC

To analyze DC biology, DC were isolated from the inguinal lymph nodes draining the tumor. Lymph nodes were excised and mashed using needles or glass slides and digested in collagenase type II and DNase for 15 min. After addition of EDTA and re-suspending, cells were applied to a filter to remove debris and DC were enriched by positive selection using anti-CD11c-labeled (N418) magnetic micro beads (Miltenyi Biotec B.V.).

To study the fate of antigen and CpG after cryo ablation, tumors were injected with fluorescently-labeled OVA protein (OVA-Alexa488 or OVA-Alexa647, 20 µg/20 µl) and Cy5-labeled CpG (CpG-Cy). CD11c-enriched cells were incubated with Fc Block (CD16/CD32; 2.4G2) and stained with anti-CD11c (HL3), biotinylated anti-CD80 (1G10/B7) or isotype controls and streptavidin-PE, all obtained from BD Biosciences. Expression of CD80 was analyzed in OVA^+^ and OVA^−^ cells gated on CD11c^+^ cells by flow cytometry (FACS Calibur, Becton Dickinson & Co, San Jose, CA). It was previously reported that uptake of the model antigen OVA by bone marrow-derived DC largely depends on the mannose receptor [23]. Therefore, we repeated our experiments with BSA-Alexa488 and no differences in uptake by DC in vivo were observed between the two antigens (data not shown).

### Ex Vivo T Cell Monitoring

Cells from the spleen and tumor draining lymph nodes were stimulated for 5 hours with PMA (0.5 µg/ml)/ionomycin (0.1 µg/ml) in the presence of Brefeldin A (10 µg/ml). Cells were analyzed for CD8 and intracellular expression of IFN-γ using flow cytometry. In addition, cells were stimulated with anti-CD3 or OVA protein for 96 hours to analyze the secretion of IFN-γ in supernatant. The IFN-γ ELISA was performed according to the manufacturer's instructions (BD Biosciences).

### Antigen-Specific CTL

To study the efficacy of the differential CpG administration to induce antigen-specific CTL, spleens and tumor-draining lymph nodes were isolated ten days after ablation. Mixed cell suspensions of lymph node and spleen were plated in 24-well plates in complete IMDM supplemented with hIL2 (10 U/ml) and stimulated with irradiated (1600 rad) B16OVA cells (5*10^4^ cells/well) that had been treated overnight with r-IFN-γ (50 U/ml) to up-regulate MHC class I presentation. At day 3–4 of culture, cells were harvested and dead cell debris was removed using a Ficoll gradient. Cells were cultured in fresh 24-well plates for another 4–5 days. To visualize tumor-specific CTL, cells were stained with FITC-conjugated CD4 (L3T4) or CD8 (53–6.7) and APC-conjugated OVA Kb tetramers (Sanquin, Amsterdam, The Netherlands) and analyzed by flow cytometry.

### Statistical Analyses

Data were analyzed using a two-tailed Student's T test or one-way ANOVA for multiple comparisons with Bonferroni as post hoc test.

## Results

### Accumulation of Activated Antigen-Loaded Mature Dendritic Cells in Draining Lymph Nodes Depends on the Route of CpG

The priming of CD8^+^ CTL depends on the unique feature of DC to capture extracellular antigens and present them on MHC class I. This process, known as cross-presentation [24], can be strongly enhanced by activation of DC via TLR9 [25]. In the present study, we investigated the impact of the route of CpG administration on DC function and antitumor responses after cryo ablation.

**Figure 1:**
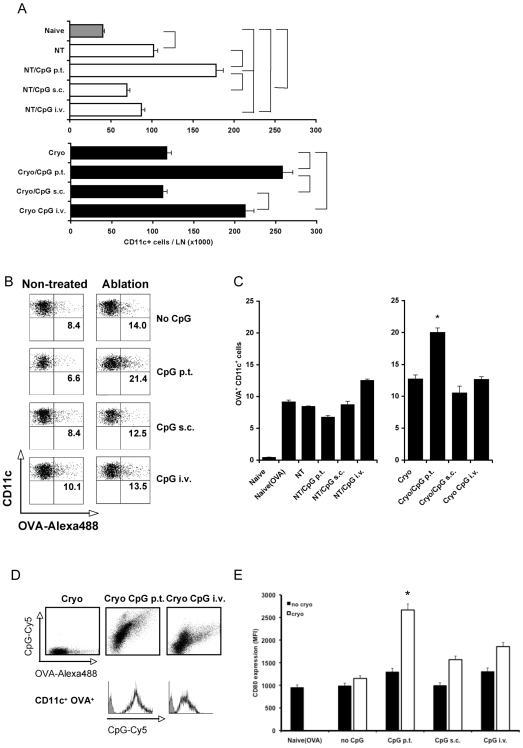
Quantity and quality of lymph node DC. (**A**) Absolute numbers of CD11c^+^ cells in the draining lymph nodes 2 days after ablation. Tumor-bearing mice were left untreated (NT) or were subjected to cryo ablation (cryo). In addition, some groups of mice received CpG via the indicated routes. Brackets indicate significant differences (p<0.05) between indicated groups (n = 4–6/group, 2 similar experiments). (**B**) Uptake of antigen after cryo ablation. OVA-Alexa488 was injected in B16OVA tumors (6–9 mm) just prior to cryo ablation to monitor the fate of antigen after in situ tumor destruction. Mice were additionally treated with CpG via the indicated routes. Two days after ablation, the uptake of antigen was analyzed in CD11c^+^ cells enriched from pools of tumor-draining lymph nodes. Numbers in the panels indicate the percentage of OVA^+^ of all CD11c^+^ cells. Data from one representative mouse is shown per group. (**C**) Quantitative analyses of replicates of experiments as shown in (B) (5 mice/group, representative of 2 experiments). (**D**) Uptake of antigen and CpG by CD11c^+^ cells monitored after p.t. and i.v. CpG-Cy5 administration and intra-tumoral injection of OVA-Alexa488. Data from one representative mouse is shown (n = 4/group). (**E**) The expression of CD80 on CD11c^+^ antigen-loaded cells. * indicates significantly different (p<0.05) from all other groups (n = 5/group, 2 similar experiments).

As shown in Figure 1A, peritumoral CpG injections markedly increased the absolute number of DC that accumulated in the tumor-draining lymph node 2 days after injection in both tumor-bearing non-treated mice and in mice subjected to cryosurgery. Subcutaneous injection of CpG at the contra lateral side did not affect the numbers of DC. Interestingly, i.v. CpG injections increased the accumulation of CD11c^+^ DC in the tumor-draining lymph node only when combined with cryo ablation (Fig. 1A).

Antigen internalization and the maturation status of DC are two major requirements for the induction of productive antigen-specific immune responses. We have previously shown that cryo ablation combined with p.t. CpG administration synergistically up-regulates the expression of co-stimulatory molecules [26]. To study whether the route of CpG administration differentially affects the ability of DC to take up antigen, OVA-Alexa-488 was injected in the tumor just prior to ablation as a surrogate marker for soluble antigens that are rapidly released from the tumor following cryosurgery [27]. Peritumoral CpG injection concurrent with ablation synergistically increased the uptake of antigen by draining lymph node DC. In contrast, both i.v. and s.c. injections failed to enhance antigen uptake in either tumor-bearing non-treated mice or mice subjected to cryosurgery (Fig. 1B–C). Applying fluorescently-labeled CpG, we additionally found that the majority of all lymph node DC from mice exposed to CpG via p.t. or i.v. injections had internalized CpG. The amount of CpG per DC was however much higher after p.t. administration (Fig. 1D) and the OVA^+^ DC had acquired increased levels of CpG compared to the OVA^+^ DC from mice that received i.v. CpG injections. Finally, s.c. and i.v. CpG injections only slightly increased CD80 expression on antigen-loaded DC, whereas p.t. CpG administration significantly enhanced the levels of co-stimulatory markers (Fig. 1E).

Together, these results demonstrate that p.t. CpG injections significantly increase the numbers of antigen- and CpG-loaded, CD80-expressing draining lymph node DC after cryo ablation in vivo. Administering CpG via the i.v. route results in the accumulation of draining lymph node DC after ablation but these DC exhibit only slightly enhanced levels of internalized antigen and CD80-expression when compared to cryo ablation alone. Subcutaneous injections of CpG at the contra lateral side do increase the number of DC in the local lymph node (data not shown), but not in the tumor-draining lymph node and fail to enhance antigen uptake and CD80 expression.

### Route of CpG Administration Determines Priming of CD4^+^ and CD8^+^ T Cells

**Figure 2:**
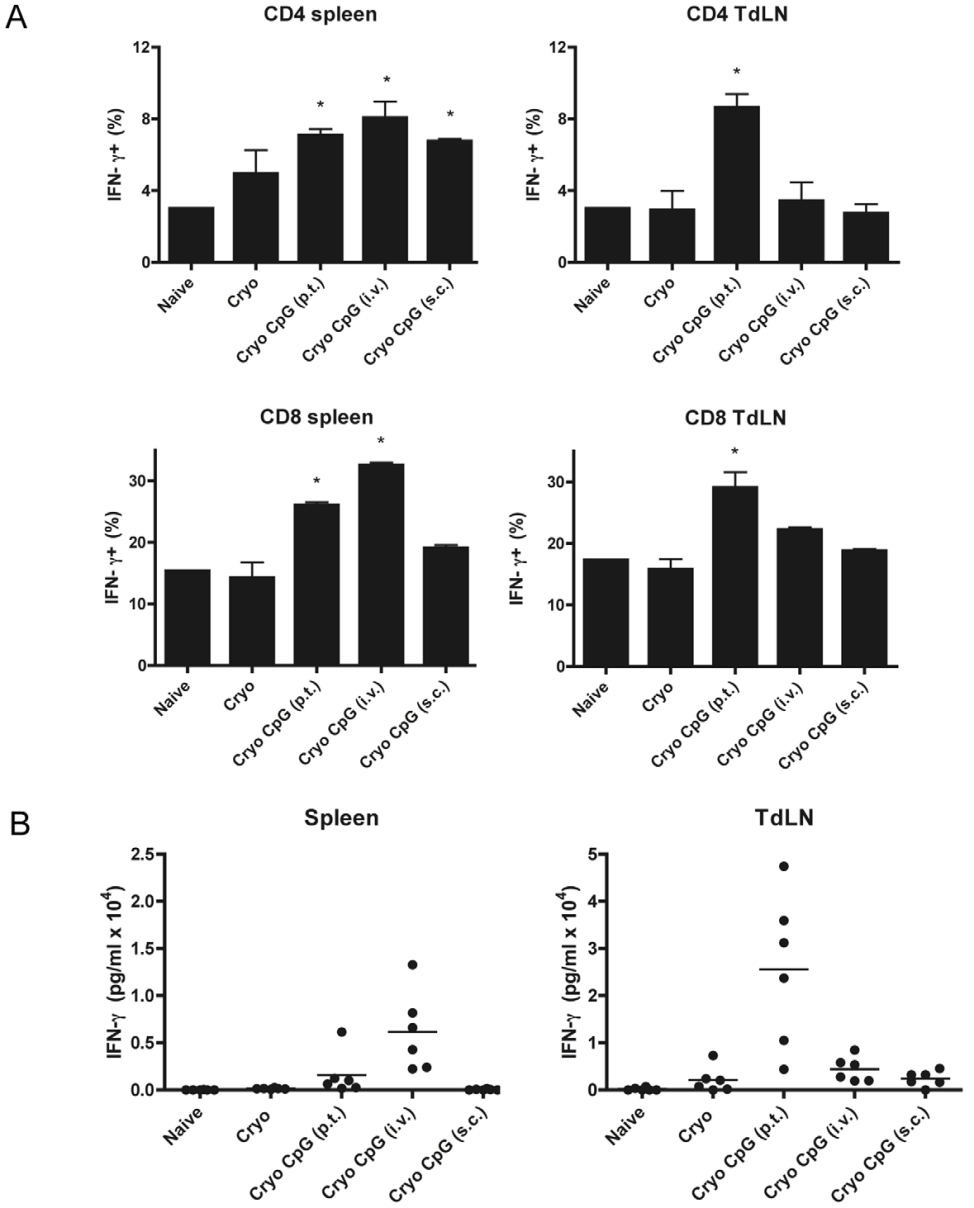
Route of CpG administration affects T cell activation. (**A**) Percentages of IFN-γ^+^ T cells within the CD4^+^ or CD8^+^ populations after cryo ablation +/− CpG. B16-OVA tumor-bearing mice were treated with cryo ablation combined with CpG administration via the indicated routes. Seven days after ablation, spleen, tumor-draining lymph nodes and non-draining lymph nodes were isolated. Cells were stimulated with PMA/ionomycin and stained for CD8, CD4 and IFN-γ. * indicate significant differences (p<0.05) between the indicated CpG-treated group and the Naïve and Cryo group. (**B**) IFN-γ production after cryo ablation +/− CpG. Cells were stimulated with OVA for 96 hours and IFN-γ was measured in supernatant by ELISA. The mean levels of the Cryo+CpG i.v. groups in both organs are significantly different from all other groups (n = 6/group, 2 experiments).

To gain further insight into the consequences of differential DC phenotypes induced by CpG administration via different routes in vivo the splenocytes and lymphocytes from the tumor draining lymph nodes were analyzed. We observed that the percentages of IFN-γ^+^ CD4^+^ and IFN-γ^+^ CD8^+^ T cells after non-specific (Fig. 2A) or specific re-stimulation (Fig. 2B), as well as the cellularity of the lymphoid organs (not shown) were affected by the CpG injection site. Peritumoral CpG injections increased IFN-γ^+^ cells predominantly in the tumor-draining lymph node and also slightly in the spleen. After i.v. injections the number of IFN-γ^+^ cells was mainly increased in the spleen. CD8^+^ IFN-γ^+^ T cells were slightly enhanced in the lymph node draining the ablated site. Subcutaneous CpG injections contra lateral of the tumor did not affect IFN-γ^+^ T cell numbers in the tumor-draining lymph node.

**Figure 3:**
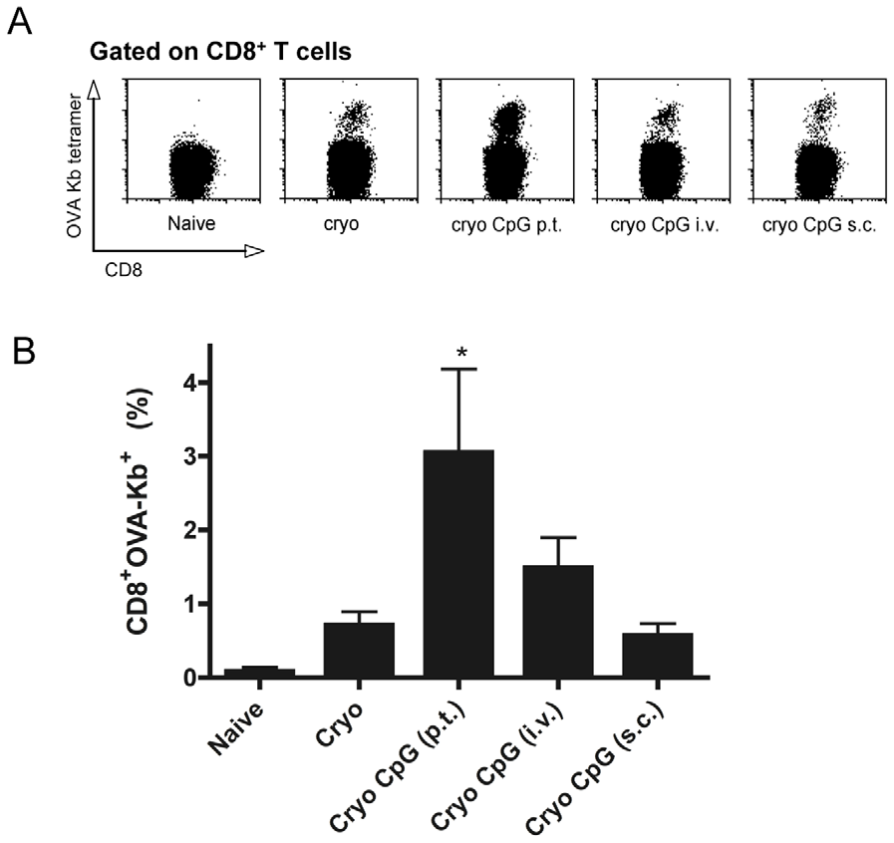
Route of CpG administration determines induction of tumor-specific CTL. (**A**) Representative dot plots of CD8^+^OVA-Kb tetramer^+^ cells of individual mice. Mice bearing B16OVA tumors (6–9 mm) were subjected to cryo ablation alone or received additional CpG injections via the indicated routes. Ten days after tumor destruction, cells from spleen and tumor draining lymph nodes were isolated and re-stimulated with IFN-γ-treated γ-irradiated B16OVA tumor cells. Cultures were cleaned by a Ficoll step after 3–4 days of culture and at day 8–9 cells were analyzed for the presence of tumor-specific CTL using APC-labeled OVA-K_b_ tetramers. Cells were gated on CD8^+^ T cells. Data from one representative mouse per group is shown. (**B**) Quantitative analyses of collective data as shown in (A) (mean levels of 2 separate experiments (4–6 mice per group/experiment)). * indicates significant differences (p<0.05) of the indicated group compared to all other groups.

Finally, enumeration of OVA-specific CD8^+^ T cells 10 days after cryosurgery confirmed the superior induction of antigen-specific CTL when CpG was administered p.t. (3.1%±1.1 of all CD8^+^, Fig. 3). Relative to cryo ablation alone (0.7%±0.2), neither i.v. nor s.c. CpG injections yielded a significant increase in CTL (1.5%±0.4; 0.60%±0.2). The same trend, although not significant, was found for CTL specific for B16 intrinsic tumor peptide TRP-2 (not shown).

Collectively, these data thus indicate that the improved loading and maturation of DC following p.t. CpG administration correlates with the observed increase in local lymphocyte activation.

### Peritumoral CpG Injections Are Superior in the Induction of Long-Term Antitumor Immunity

The aforementioned data show that the injection site of CpG relative to the ablated tumor area is a crucial determinant in the induction of tumor-specific CTL responses. To further investigate its importance, tumor-bearing mice were treated with cryo ablation and CpG was given either p.t., s.c. or i.v. Forty days after ablation, tumor-free mice were challenged with a lethal dose of tumor cells.

**Figure 4:**
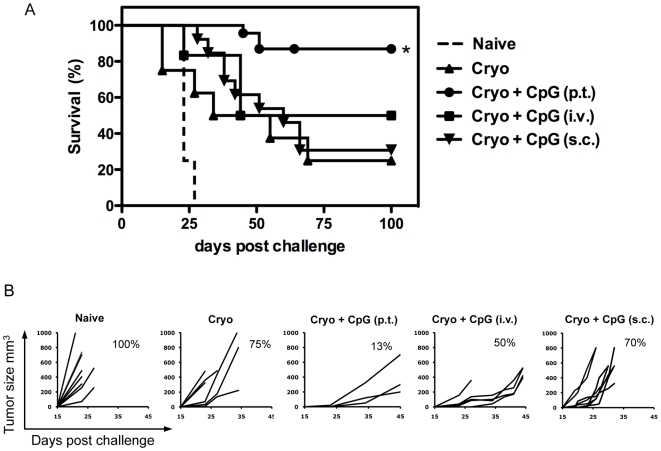
Route of CpG administration determines efficacy of antitumor immunity. (**A**) Kaplan-Meier survival curves of naïve mice versus mice that have been treated with cryo ablation alone, or in combination with concurrent CpG administration via the indicated routes. Established B16OVA tumors on the right femur were treated with cryo ablation alone or in combination with CpG administrations via different routes: p.t., i.v., or s.c. contra lateral of the tumor. Forty days later, naïve and tumor-free mice (8–13 mice per group) received a s.c. re-challenge with tumor cells (25.000 B16OVA cells). (**B**) Tumor size determined every 2–3 days after treatment. Data is representative of two independent experiments.

Cryosurgery combined with p.t. CpG administrations protected ∼90% of all mice, whereas s.c. and i.v. CpG injections failed to significantly improve the responses induced by cryosurgery alone (Fig. 4A). Although i.v. CpG administration failed to increase survival, the tumor growth was considerably slower than in mice treated with s.c. injections or with cryo ablation alone (Fig. 4B). These data indicate that i.v. CpG injections may induce antitumor responses that are able to limit tumor growth and prolong survival to some extent. Nevertheless, when mice received lethal tumor challenges i.v. CpG injections protected only 50% of the mice, whereas in case of p.t. CpG injections ∼90% of the mice survived. CpG pre-treatment of naive mice did not provide any protection against lethal tumor challenges irrespective of the administration route (data not shown).

**Figure 5:**
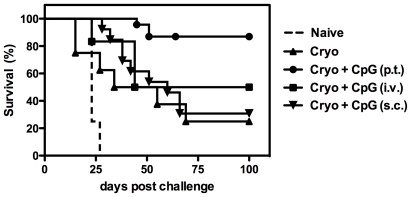
Timing of i.v. CpG injections determines antitumor immunity. Established B16OVA tumors were treated with cryo ablation alone or in combination with i.v. CpG administration concurrent with ablation, or 1 day before or after ablation. Forty days later, naïve and treated tumor-free mice (7–10 mice per group) received a s.c. re-challenge with tumor cells (25.000–50.000 B16OVA cells) on the contra-lateral flank and survival was monitored. Representative of 2 experiments is shown.

We have previously shown that the timing of CpG administration is critical for the efficacy of p.t. CpG injections when combined with ablation. The reduced efficacy of i.v. administered CpG in the current experiments may therefore relate to the delay in which CpG-ODN reach the released antigens at the ablation site. CpG was therefore injected 1 day before, concurrent with, or 1 day after ablation. Administration of CpG 1 day before ablation resulted in similar results as observed for CpG injections concurrent with ablation (40–45% of the mice survived). In contrast, CpG administration 1 day after ablation failed to enhance antitumor immunity as induced by cryo ablation alone (Fig. 5). The reduced efficacy of i.v. CpG administrations compared to p.t. exposure is thus not due to timing aspects.

## Discussion

Although mouse models have clearly shown the potent antitumor effects of CpG, clinical results have so far been disappointing, with recent phase 2 and 3 trials in non-small cell lung cancer being dropped due to the failure of s.c. CpG-ODN administration to improve clinical outcome [28]. In the present study, we show that the route of administration determines the efficacy of CpG to stimulate immune responses after in situ tumor destruction. Peritumoral CpG administration increases the numbers of activated, mature, antigen-bearing DC in the tumor-draining lymph node and stimulates the formation of tumor-specific CTL leading to superior memory responses against tumor antigens. Subcutaneous CpG injections distant from the tumor fail to enhance antitumor immunity. Also systemic CpG administration through i.v. injection only provides limited protection (50%) as compared to peritumoral injection (90% protection).

Applying a novel clinically relevant in situ tumor destruction model, we have previously reported that the failure or success of CpG as a vaccine adjuvant depends on the co-localization of antigen and CpG within endosomal DC compartments and hence on the timing of CpG administration. Dendritic cells were only able to cross-present antigens and promote CTL induction in conditions where antigen and CpG were co-localized within DC. In fact, p.t. CpG administration 1 day before or after tumor ablation, instead of concomitant CpG injection, already had a major impact on its efficacy [21]. Based on these findings, we set out to determine the effect of the route of administration on CpG in this model system. While s.c. CpG injections distant from the tumor fail to initiate any antitumor immunity, i.v. CpG administration yielded some survival benefit (not significant) over cryo ablation alone. These differences in the efficacy of CpG did not relate to differences in timing, as different timing regimens for i.v. CpG administration did not improve its efficacy. These data show that both the time and route of CpG administration determine the efficacy of CpG therapy and imply that the availability of CpG in time and place in (lymphoid) tissues is an important factor in the adjuvanticity of CpG.

It has previously been postulated that the outcome of CpG-ODN administration via a specific route may depend on the type of cells that are encountered by the ODN at a particular anatomical site [29]. The responsive cells in the lymph nodes draining the injection site may activate a different pathway for TLR9 signaling than cells in the spleen. In agreement with this hypothesis are the observations that intra-tumoral [25], p.t. [26], [30] or intra-lymphatic [31] administration of CpG showed the anticipated Th1-promoting effect and were highly efficient in improving T cell-mediated immunity. In contrast, systemic (i.v. or i.p.) application of CpG resulted in T cell suppression rather than immune activation [32], [33], [34]. Similar indications came from human trials in which s.c. administrations of antigen plus CpG induced a systemic Th1-like cytokine expression in serum, whereas intravenous injections caused no such effects [35].

Here we show that DC taken from the tumor-draining lymph node after i.v. CpG exposure have internalized suboptimal levels of antigen and fail to express high levels of co-stimulatory molecules. This may be due to the inability of incoming DC to internalize or transfer antigen after a previous encounter with CpG. As both resident DC and migrating DC are required for the induction of productive CTL priming [36] this may limit the induction of functional CTL. In addition, it was previously shown that i.v. administration induced expression of indoleamine 2,3-dioxygenase (IDO) in DC, an enzyme linked to immune regulation and formation of regulatory T cells (Treg), leading to suppressed T cell expansion and CTL activity. Although we did not investigate the immune regulatory properties of DC directly, we observed significantly increased numbers of CD4^+^Foxp3^+^ Treg in the spleen of mice treated i.v. with CpG (data not shown). Treg hamper the induction of antitumor immune responses and are known to be regulated via multiple mechanisms [37]. The observed sub-optimal maturation of DC in the tumor draining lymph node combined with the induction of IDO-expressing DC in the spleen following i.v. CpG administration may prevent optimal immune activation.

The present study emphasizes that in cancer immunotherapy CpG should be administered in close proximity of the released tumor antigens. In clinical studies CpG is generally administered independent of tumor antigen release [38]. From a clinical point of view it is preferred to administer CpG at an easy accessible location and in large amounts according to the rationale that it will travel to the tumor location. However, we show here that neither s.c. administration on a distant site or i.v. injection fully exploit the adjuvant capacity of CpG. In line with our data showing that co-localization of antigen and CpG favours immunity, potent immune stimulatory responses of CpG were found also in humans when antigen (recombinant hepatitis B surface antigen) and CpG (CPG 7909) were co-injected [39].

It would be interesting to see if the findings presented here also apply to other situations of tumor cell death, like that in chemotherapy or radiation. Nevertheless, as both cryosurgery, and other in situ tumor destruction treatments (e.g. radiofrequency ablation or high-intensity focussed ultrasound) are applied in patients, the combinational therapy with CpG as described in this paper is clinically relevant. The instantaneous availability of tumor antigens and destruction of the majority of tumor tissue with ablation treatments may provide a scenario in which the effects of CpG can be appreciated even in advanced disease. Our data emphasize that the location of CpG administration should be an imperative element in the design of future clinical trials.
